# Digital Exposure and Skin Health in Children and Adolescents: A State-of-the-Art Review

**DOI:** 10.7759/cureus.110340

**Published:** 2026-06-06

**Authors:** Collin Yee, Rasya Reddy, Razia Sultana, Zeliha Karabudak Ozbakir, Chineche R Anigbo, Ainaz Kolesnikova, Shreeya Subba Rai, Sofia L Rivera-Torres, Manju Rai

**Affiliations:** 1 Internal Medicine, Tulane University School of Medicine, New Orleans, USA; 2 Internal Medicine, Kasturba Medical College, Manipal, Manipal, IND; 3 Internal Medicine, Anwer Khan Modern Medical College, Dhaka, BGD; 4 Internal Medicine, Gazi University Hospital, Ankara, TUR; 5 Dermatology, Enugu State University Teaching Hospital, Enugu, NGA; 6 Internal Medicine, Kazan Federal University (KFU) Institute of Fundamental Medicine and Biology, Tatarstan, RUS; 7 Internal Medicine, Central American Health Sciences University, Ladyville, BLZ; 8 Internal Medicine, Universidad de Monterrey (UDEM), San Pedro Garza García, MEX; 9 Biotechnology, Shri Venkateshwara University, Gajraula, IND

**Keywords:** acne vulgaris, atopic dermatitis, blue light, circadian rhythm disruption, digital exposure, lifestyle factors, pediatric dermatology, psychodermatology, screen time, skin barrier dysfunction

## Abstract

The rapid expansion of digital technology has led to unprecedented levels of screen exposure among children and adolescents, raising concerns regarding its potential impact on dermatologic health. This state-of-the-art review aims to synthesize current evidence regarding the pathophysiological mechanisms, clinical manifestations, and preventive strategies associated with digital exposure in pediatric populations. A comprehensive literature search was conducted across PubMed, Scopus, and Google Scholar from inception through January 2026 using terms related to digital exposure, screen time, blue light, pediatric dermatology, circadian rhythm disruption, and psychodermatology. Evidence from experimental, clinical, and epidemiological studies was thematically analyzed. Available evidence suggests that excessive digital exposure may influence skin health through multiple interconnected mechanisms, including reactive oxygen species-mediated oxidative stress, mitochondrial dysfunction, circadian rhythm disruption, impaired skin barrier integrity, and neuroendocrine activation. Indirect lifestyle factors associated with prolonged screen use, such as sleep disturbance, psychological stress, sedentary behavior, unhealthy dietary patterns, poor hydration habits, and reduced outdoor activity, may further contribute to systemic inflammation and metabolic dysregulation. Pediatric skin, characterized by an immature epidermal barrier, evolving immune responses, and heightened sensitivity to environmental stimuli, appears particularly susceptible to these cumulative effects. Clinically, digital exposure has been linked to a spectrum of dermatologic conditions, including acne vulgaris, atopic dermatitis exacerbations, contact dermatitis, frictional dermatoses, xerosis associated with prolonged screen use and inadequate hydration, pigmentary disorders, and lifestyle-associated metabolic changes that may indirectly affect dermatologic outcomes. Psychodermatologic interactions and social media-driven behaviors may additionally influence disease perception and management. Current evidence remains heterogeneous and largely associative; however, the convergence of mechanistic and epidemiological findings highlights digital exposure as an emerging environmental factor in pediatric dermatology. Further longitudinal studies are needed to clarify causal relationships, evaluate long-term outcomes, and develop evidence-based preventive strategies that support healthy digital engagement while minimizing dermatologic risk.

## Introduction and background

The rapid expansion of digital technology has fundamentally transformed the daily lives of children and adolescents worldwide. The use of screen-based devices, including smartphones, tablets, computers, and televisions, has increased substantially over the past decade, with an additional surge observed during the COVID-19 pandemic. During this period, approximately 59% of children reported increased recreational screen time, with particularly pronounced rises among younger age groups, averaging over 1.5 hours of additional daily exposure [1,2]. These trends reflect a broader global shift toward digitally mediated education, entertainment, and social interaction.

International health organizations have raised concerns regarding excessive screen exposure in pediatric populations. The World Health Organization (WHO) recommends limiting sedentary screen time in young children to promote optimal physical and developmental health. Similarly, the American Academy of Pediatrics (AAP) advises restricting recreational screen time to ≤2 hours per day for older children and adolescents, while the United Nations Children's Fund (UNICEF) reports a rapid global increase in digital access among youth [3]. These data underscore the scale and urgency of understanding the health implications of digital exposure.

Digital exposure should be conceptualized as a novel, multifactorial environmental determinant of dermatologic health, integrating physical, behavioral, and psychosocial pathways [4]. The skin, as the body's primary interface with the external environment, is uniquely susceptible to such influences [3,4]. Emerging evidence suggests that prolonged interaction with digital devices may contribute to a spectrum of dermatologic effects, mediated through both direct and indirect mechanisms [5-7].

From a photobiological perspective, electronic devices emit visible blue light, which may influence cutaneous homeostasis through oxidative and barrier-related pathways [8-14]. Experimental data suggest that blue light exposure may induce pigmentary changes and contribute to photoaging, although its clinical significance relative to solar radiation remains under investigation [5,8,9]. In addition to light exposure, device use has been linked to mechanical and environmental factors, such as friction, pressure, and heat, contributing to conditions including acne mechanica, contact dermatitis, and localized irritation [6,7,15,16].

Beyond direct cutaneous effects, digital exposure influences dermatologic health indirectly through behavioral and lifestyle pathways. Excessive screen time is strongly associated with sleep disturbances, including delayed sleep onset and reduced sleep duration [17]. Sleep and circadian disturbances associated with screen use may adversely affect skin recovery and inflammatory regulation [8,18]. Furthermore, increased sedentary behavior and unhealthy dietary patterns linked to prolonged screen use contribute to obesity and metabolic dysregulation, which are recognized modifiers of dermatologic diseases such as acne vulgaris, psoriasis, and hidradenitis suppurativa [3,19-21].

Reduced outdoor activity represents another important behavioral consequence of excessive digital engagement. Increased screen time may contribute to lower sunlight exposure and reduced outdoor activity, which, together with dietary, environmental, and individual factors, can predispose children to vitamin D deficiency, a condition associated with several inflammatory and autoimmune skin disorders [20,22]. Concurrently, the rise of social media platforms introduces additional psychosocial dimensions, including the dissemination of skincare misinformation and altered health-seeking behaviors, which may further influence dermatologic outcomes [23,24].

While emerging mechanistic evidence provides insight into the biological pathways linking digital exposure to dermatologic health, understanding the scale and distribution of this exposure at a population level is equally critical [23]. Epidemiological data offer valuable context by characterizing patterns of digital device use across age groups, behavioral profiles, and sociocultural settings, thereby informing the potential burden of associated dermatologic outcomes [23,24].

This state-of-the-art review synthesizes emerging evidence and proposes an integrated framework linking digital exposure to dermatologic health in pediatric populations. This review aims to (1) elucidate the pathophysiological mechanisms linking digital exposure to dermatologic health in children and adolescents, (2) summarize the spectrum of clinical dermatologic manifestations associated with these exposures, and (3) discuss current and emerging preventive strategies. The review is structured into sections addressing mechanistic pathways, clinical implications, and evidence-based approaches to mitigation.

## Review

Methodology

This narrative review was conducted to synthesize current evidence on the impact of digital exposure on dermatologic health in children and adolescents. A comprehensive literature search was performed across electronic databases, including PubMed, Scopus, and Google Scholar, from inception through January 2026.

Search terms were developed to capture key domains of interest and included combinations of "digital exposure", "screen time", "blue light", "high-energy visible light", "pediatric dermatology", "acne", "atopic dermatitis", "skin barrier", "circadian rhythm", "psychodermatology", and "lifestyle factors". Boolean operators (AND/OR) were applied to refine the search strategy.

Studies were identified and selected based on their relevance to the scope and objectives of the review, with emphasis placed on peer-reviewed original articles, systematic reviews, and clinically or mechanistically relevant evidence. Preference was given to recent literature and studies with significant relevance to pediatric dermatology and digital exposure. Additional articles were identified through manual screening of reference lists. Both pediatric-focused studies and selected adult studies with strong mechanistic relevance were included to provide a comprehensive perspective.

**Figure 1:**
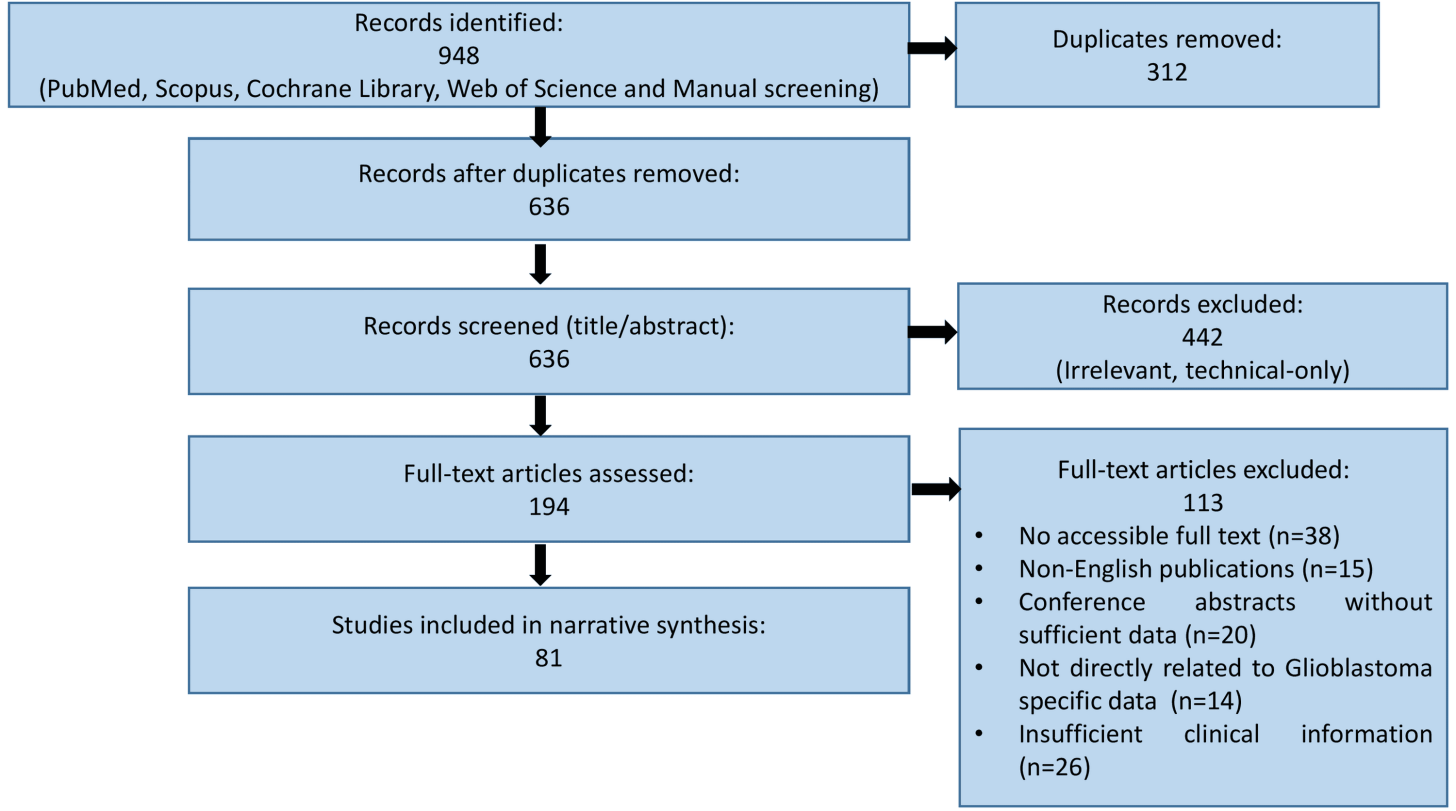
Structured Flow Diagram of Literature Identification and Study Selection Process The figure was created using PowerPoint (Microsoft® Corp., Redmond, WA)

Although this review was narrative in design, a structured literature search and transparent study selection approach were adopted to improve methodological rigor and clarity in evidence synthesis. A structured flow diagram summarizing the literature identification and article selection process is presented in Figure 1.

As this is not a systematic review, formal inclusion and exclusion criteria, risk-of-bias assessment, and quantitative synthesis were not performed. Instead, included studies were critically appraised for methodological quality, scientific rigor, and relevance. Evidence was synthesized thematically into key domains, including epidemiology, cutaneous biology, pathophysiological mechanisms, clinical manifestations, and preventive strategies. Given the narrative design and heterogeneity of the available literature, no formal quantitative synthesis or meta-analysis was performed, and conclusions were derived from qualitative thematic interpretation of mechanistic, observational, and clinical evidence.

Epidemiology of digital device use and associated dermatologic concerns 

Building upon the growing recognition of digital exposure as a determinant of dermatologic health, the global proliferation of digital devices has led to near-universal exposure among children and adolescents. Epidemiological studies indicate that school-aged children (6-14 years) spend an average of approximately three hours per day on screen-based activities, with higher durations reported in urban and high-income settings [25]. Contributing factors include the presence of digital devices in bedrooms, reduced outdoor activity, and increasing reliance on screens for both education and entertainment [26]. Parental concerns regarding the behavioral and health impacts of screen exposure have also risen significantly in recent years [27].

The COVID-19 pandemic further accelerated these trends, with multiple studies demonstrating a substantial increase in recreational screen time among children and adolescents compared to pre-pandemic levels [28]. This shift has persisted beyond the pandemic period, suggesting a sustained change in digital behavior. Concurrently, increased screen exposure has been associated with a range of adverse health outcomes, including obesity, sleep disorders, and mental health disturbances, which may indirectly influence dermatologic health [27].

Age-specific patterns of digital exposure reveal important distinctions. In toddlers and preschool-aged children, screen use is often passive and mediated by caregivers, frequently employed as a behavioral management tool. The WHO recommends no screen exposure for infants and limiting use to less than one hour per day for children aged one to five years [29]. School-aged children exhibit a transition toward mixed educational and recreational use, while adolescents demonstrate the highest levels of exposure, driven largely by social media engagement, gaming, and digital communication. This progression is accompanied by increasing autonomy, raising concerns regarding excessive and unsupervised use.

Emerging epidemiological evidence suggests a growing association between digital device use and dermatologic conditions, although data remain limited. Device-related dermatoses, such as acne mechanica ("cell-phone acne"), frictional dermatitis, and contact dermatitis due to metal allergens (e.g., nickel, cobalt), have been increasingly reported, particularly in areas of prolonged skin-device contact [30]. Repetitive mechanical stress from gaming and smartphone use has also been linked to localized conditions such as "gamer's thumb" and palmar hyperkeratosis [30]. While large-scale population-based studies are scarce, these findings highlight an evolving pattern of technology-associated skin disorders.

Gender differences in digital behavior further influence exposure patterns and associated health outcomes. Epidemiological studies suggest that males are more likely to engage in gaming-related activities, whereas females demonstrate higher engagement with social media platforms [31,32]. These differences may have dermatologic implications, including variations in exposure to frictional forces, cosmetic product use influenced by online content, and psychosocial stressors affecting skin conditions such as acne and atopic dermatitis. Such patterns underscore the importance of gender-sensitive approaches in both research and intervention strategies.

Digital addiction and sedentary lifestyle factors represent critical mediators linking screen exposure to dermatologic health. Excessive smartphone use has been associated with reduced physical activity, poor sleep hygiene, and unhealthy dietary behaviors, all of which contribute to systemic inflammation and metabolic dysregulation [26,33]. These factors are recognized contributors to the exacerbation of chronic inflammatory skin diseases. Furthermore, high rates of behavioral addictions, including internet addiction (29.7%) and substance use, have been reported among patients with chronic dermatologic conditions, suggesting a bidirectional relationship between digital behaviors and skin health [34].

Collectively, these epidemiological patterns reinforce the need to consider digital exposure within the broader framework of environmental determinants of skin health in pediatric populations. Although current evidence linking digital device use to specific skin outcomes remains limited and heterogeneous, the convergence of behavioral, environmental, and psychosocial factors warrants further investigation through large-scale, longitudinal studies.

Cutaneous biology in children and adolescents

Pediatric skin differs structurally and functionally from adult skin, conferring distinct biological properties that influence its response to environmental exposures. In early life, the epidermis is thinner, with a less compact stratum corneum, reduced corneocyte cohesion, and incomplete lipid organization. These features contribute to increased transepidermal water loss (TEWL) and enhanced percutaneous absorption, reflecting an inherently less robust barrier function [35,36]. Importantly, skin barrier maturation is a prolonged process extending into late childhood, during which key structural components, including filaggrin, ceramides, and natural moisturizing factors, gradually reach adult levels [35]. This developmental immaturity renders pediatric skin more susceptible to external stressors, including emerging digital environmental exposures.

Adolescence introduces additional complexity through hormonally driven changes in skin physiology. The rise in androgens, including dehydroepiandrosterone sulfate and testosterone, stimulates sebaceous gland activity, increasing sebum production and altering follicular keratinization [37]. These changes create a microenvironment conducive to acne development. Notably, hormonal influences interact with external triggers, including psychosocial stress and sleep disruption, both of which are prevalent in digitally engaged adolescents. Stress-related neuroendocrine activation may further amplify inflammatory signaling and sebaceous activity [37].

The pediatric immune system also contributes to heightened cutaneous vulnerability. In early life, immune responses are relatively skewed toward a T-helper 2 phenotype, accompanied by reduced expression of antimicrobial peptides and an immature innate immune response [38]. This immunologic profile underlies the increased prevalence of inflammatory skin conditions, such as atopic dermatitis. Furthermore, cutaneous immune function is closely regulated by circadian rhythms, which coordinate processes including barrier repair, cytokine balance, and cellular turnover. Disruption of these regulatory systems may predispose pediatric skin to inflammation and impaired recovery following environmental insults [37,38].

A defining feature of pediatric skin biology is its reduced resilience to oxidative and phototoxic stress. Compared with adults, children have lower levels of endogenous antioxidants and less cumulative photoadaptation, limiting their ability to neutralize reactive oxygen species (ROS) generated by environmental exposures [36,39]. In this context, repeated low-level exposures, such as those associated with prolonged digital device use, may exert cumulative effects over time. Rather than acting through a single pathway, these exposures may amplify existing biologic vulnerabilities and impair skin resilience over time.

Circadian regulation plays an important role in epidermal repair, immune balance, and barrier recovery. In children and adolescents, sleep disruption associated with excessive screen exposure may impair these restorative processes and increase susceptibility to inflammatory skin conditions [40,41].

The interaction between developmental biology and environmental exposure is further influenced by behavioral factors characteristic of childhood and adolescence. Reduced outdoor activity, irregular sleep patterns, and heightened psychosocial stress, frequently associated with high levels of digital engagement, intersect with intrinsic biological vulnerabilities to influence skin health. These interactions do not act in isolation but rather converge across multiple pathways, including barrier dysfunction, immune dysregulation, and neuroendocrine activation.

Collectively, these features underscore a central concept: pediatric skin is not merely a smaller version of adult skin, but a dynamic and evolving organ system with unique susceptibilities. The combination of an immature epidermal barrier, hormonally driven changes in adolescence, a developing immune system, and heightened sensitivity to environmental cues creates a biological context in which digital environmental factors may exert amplified effects. Understanding these age-specific characteristics is essential for interpreting the dermatologic implications of digital exposure and for developing targeted preventive and therapeutic strategies in pediatric populations. The mechanistic pathways linking digital screen exposure to dermatologic vulnerability in pediatric populations are summarized in Table 1.

**Table 1:** Mechanistic Pathways Linking Digital Screen Exposure to Dermatologic Vulnerability in Pediatric and Adolescent Skin Abbreviations: HEV, high-energy visible; ROS, reactive oxygen species; GSH, glutathione; SOD, superoxide dismutase; MMP-1, matrix metalloproteinase-1; TGF-β, transforming growth factor-beta; JNK, c-Jun N-terminal kinase; EGFR, epidermal growth factor receptor; BMAL1, brain and muscle ARNT-like 1; TEWL, transepidermal water loss; AD, atopic dermatitis; HPA, hypothalamic-pituitary-adrenal; CRH, corticotropin-releasing hormone; DHEA-S, dehydroepiandrosterone sulfate; IGF-1, insulin-like growth factor-1; NREM, non-rapid eye movement; UV-B, ultraviolet B

Pathway	Pediatric Vulnerability Factor	Mechanism of Digital Exposure Impact	Clinical Consequence
Blue Light (HEV)	Thinner epidermis; lower antioxidant reserves (GSH, SOD); reduced melanin in younger children	Flavin-mediated ROS generation (superoxide); MMP-1 activation via TGF-β/JNK/EGFR; opsin-3–mediated melanogenesis	Photoaging; collagen degradation; hyperpigmentation (Fitzpatrick III-V); cumulative DNA damage
Circadian Disruption/Melatonin Suppression	Heightened melatonin sensitivity to evening light in adolescents; immature circadian entrainment	Screen-emitted blue light suppresses pineal melatonin; disrupts BMAL1/Period2 clock genes in skin cells	Impaired nocturnal skin repair; reduced filaggrin/TEWL recovery; Th2-skewed inflammation; AD exacerbation
Sleep Disruption → Barrier Dysfunction	Ongoing barrier maturation (filaggrin, ceramides not at adult levels); Th2-dominant immunity	Screen-induced sleep-onset delay and fragmentation; reduced NREM sleep; elevated cortisol	Impaired barrier repair; systemic inflammation; AD and eczema flares; microbiome dysbiosis
Psychological Stress → Neuroendocrine Activation	HPA axis lability during puberty; androgenic surge (DHEA-S, testosterone, IGF-1)	Screen-mediated anxiety, social comparison, and social media stress; CRH-driven sebocyte activation	Amplified sebum production; follicular inflammation; acne vulgaris exacerbation
Vitamin D Deficiency (Behavioral)	High screen time displaces outdoor activity; rapid skeletal growth demands	Reduced UV-B-mediated cutaneous cholecalciferol synthesis; ≥5 h/day screen time doubles deficiency risk	Weakened epidermal innate immunity; impaired wound healing; increased susceptibility to infections

Pathophysiological mechanisms linking digital exposure to skin damage

Digital exposure represents a complex environmental stimulus capable of influencing cutaneous biology through multiple interconnected molecular pathways. Unlike traditional environmental stressors, such as ultraviolet radiation, digital exposures are characterized by chronic, low-intensity, and repetitive stimuli, necessitating a mechanistic framework that integrates photobiological, biochemical, and biophysical processes.

Blue Light (High-Energy Visible (HEV) Light) Exposure

Electronic devices emit HEV light, particularly within the blue spectrum (approximately 400-500 nm), which penetrates the skin to the level of the dermis. Although the intensity of device-emitted blue light is lower than that of solar radiation, cumulative exposure may have biologically relevant effects. Experimental studies demonstrate that HEV light can initiate photochemical reactions within cutaneous cells, acting as a trigger for downstream oxidative stress pathways [42].

ROS and Oxidative Stress Pathways

A central mechanism underlying digital exposure-induced skin damage is the generation of ROS. Blue light interacts with endogenous chromophores, leading to the formation of ROS, including superoxide anions and hydrogen peroxide [43,44]. While ROS play physiological roles in cellular signaling, excessive accumulation disrupts redox homeostasis, resulting in oxidative stress. This imbalance activates signaling cascades that upregulate matrix metalloproteinases, degrade extracellular matrix components such as collagen, and promote inflammatory responses, collectively contributing to premature skin aging and structural damage [45].

Mitochondrial Dysfunction and DNA Damage

Increased ROS production can impair mitochondrial respiratory chain function, reduce adenosine triphosphate (ATP) synthesis, and promote mitochondrial DNA (mtDNA) damage [44]. These alterations compromise cellular energy metabolism and enhance apoptotic signaling pathways. In addition, oxidative stress may induce nuclear DNA damage, including strand breaks and base modifications, further impairing cellular repair mechanisms. Over time, these molecular events contribute to cumulative cellular dysfunction and may accelerate cutaneous aging processes.

Circadian Rhythm Disruption and Impaired Skin Repair

Cutaneous physiology is tightly regulated by circadian rhythms, which coordinate processes such as cell proliferation, DNA repair, and barrier restoration. Exposure to artificial light, particularly during evening hours, disrupts circadian signaling by suppressing melatonin secretion and altering clock gene expression [46]. This disruption impairs nocturnal skin repair mechanisms, reduces antioxidant defenses, and promotes a pro-inflammatory microenvironment. At the molecular level, altered circadian regulation has been associated with impaired keratinocyte proliferation, delayed wound healing, and dysregulation of immune responses, thereby contributing to skin vulnerability.

Impact on Skin Barrier Function

The integrity of the skin barrier is critical for maintaining hydration and protecting against environmental insults. Emerging evidence suggests that digital exposure may compromise barrier function through multiple mechanisms. Oxidative stress can disrupt lipid organization within the stratum corneum, impair filaggrin expression, and increase TEWL. These alterations collectively weaken the skin's defensive capacity, increasing susceptibility to irritation, inflammation, and chronic dermatologic conditions.

Thermal Effects from Prolonged Device Contact

Prolonged contact with digital devices, particularly laptops and smartphones, can generate localized thermal effects. Although typically mild, repeated heat exposure may contribute to cutaneous changes through sustained vasodilation and increased metabolic activity. Chronic low-grade thermal exposure has been implicated in conditions such as erythema ab igne and may exacerbate pigmentary changes through melanocyte stimulation [45]. While the clinical significance of these effects in routine device use remains under investigation, they represent a plausible contributory pathway in digitally mediated skin damage.

Electromagnetic Fields (EMFs): Controversial and Inconclusive Evidence

Digital devices also emit low-level EMFs, raising questions regarding their potential biological effects on the skin. Current evidence, however, remains limited and inconclusive. Experimental studies, including animal models, have suggested possible alterations in oxidative stress markers and trace element levels following prolonged EMF exposure [47]. However, these findings are not consistently replicated in human studies, and their relevance to clinical dermatology remains uncertain. Consequently, EMF-related skin effects should be interpreted with caution, and further high-quality research is required to establish any definitive associations.

**Figure 2:**
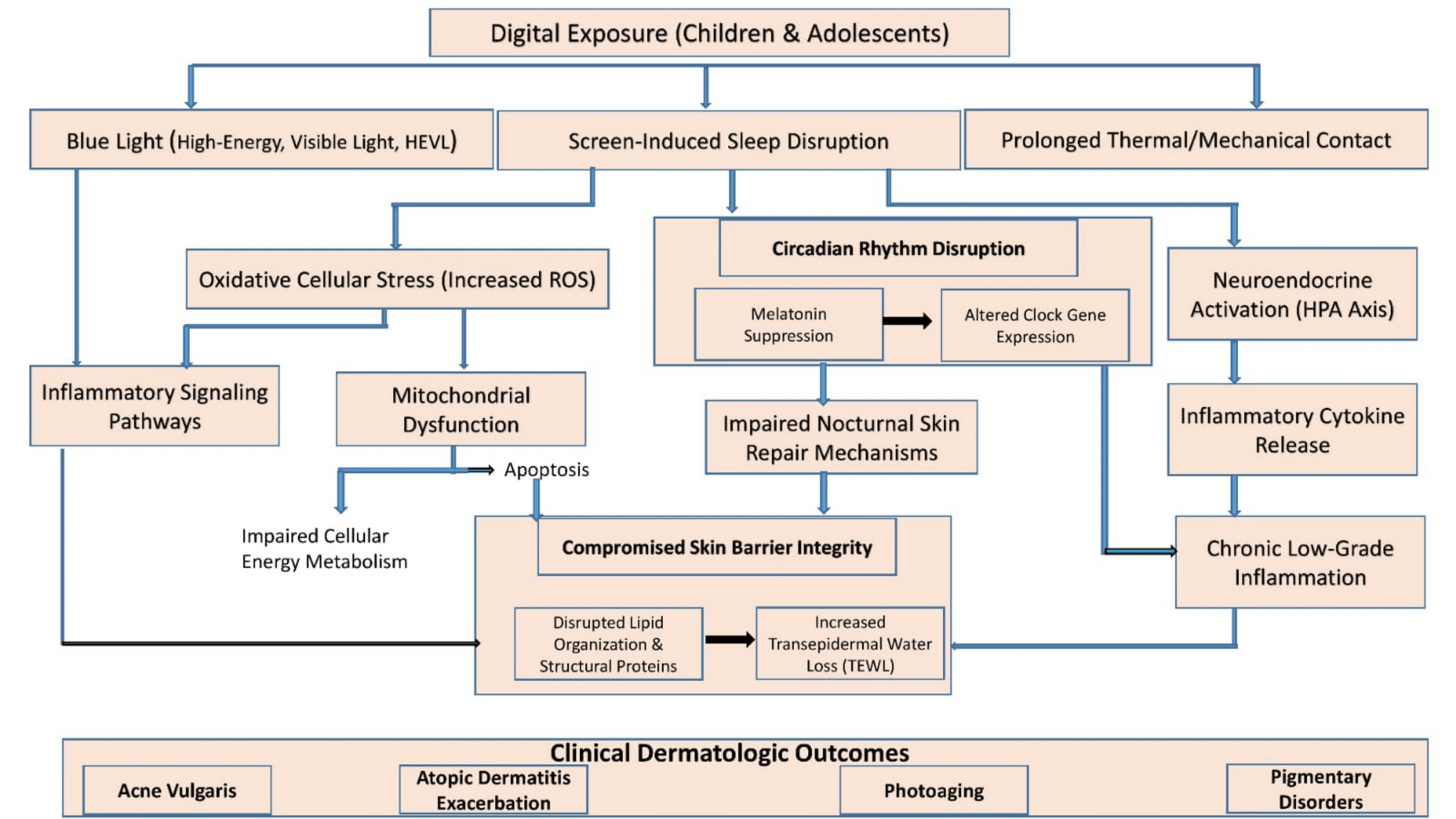
Integrated Mechanistic Pathways Linking Digital Exposure to Cutaneous Damage in Pediatric Skin The figure was created by authors in Biorender.com.

Collectively, these mechanisms illustrate that digital exposure exerts its effects on the skin through a convergence of oxidative, circadian, and barrier-related pathways. Rather than acting in isolation, these processes interact dynamically, amplifying cellular stress and impairing the skin's capacity for repair and regeneration. Understanding these molecular mechanisms provides a critical foundation for interpreting the clinical dermatologic manifestations associated with digital exposure and for developing targeted preventive strategies. The integrated mechanistic pathways linking digital exposure to cutaneous damage are illustrated in Figure 2.

Device-related dermatologic conditions 

The widespread and sustained use of smartphones, tablets, and gaming devices has led to increasing recognition of dermatologic conditions arising at the device-skin interface. These disorders result from localized interactions between the skin and digital devices, where mechanical forces, occlusion, heat, and exposure to allergens converge to disrupt the cutaneous microenvironment. In pediatric and adolescent populations, characterized by prolonged daily device use and heightened behavioral engagement, these conditions are increasingly encountered in clinical practice.

Allergic Contact Dermatitis (“Cell Phone Dermatitis”)

Allergic contact dermatitis related to digital devices is most commonly attributed to nickel and, less frequently, cobalt, present in metallic components of smartphones and tablets. Nickel remains one of the most prevalent contact allergens globally, and sensitization is particularly common in adolescents [48]. Repeated exposure to nickel-containing surfaces can trigger a type IV delayed hypersensitivity reaction, manifesting as erythema, pruritus, vesiculation, and scaling at sites of contact, including the cheek, ear, jawline, and fingers [49].

Recent reports suggest that device-related nickel dermatitis is increasingly recognized, particularly in individuals with prolonged daily exposure exceeding several hours [50]. Pediatric patients may be especially vulnerable due to frequent device handling and closer skin-device contact. Diagnosis is typically confirmed by patch testing, and management involves minimizing exposure through protective cases, avoidance of direct metal contact, and use of hypoallergenic device materials.

Frictional Dermatoses

Frictional dermatoses are among the most characteristic manifestations of device-related skin injury. Repetitive mechanical stress from prolonged texting, gaming, or scrolling can lead to localized skin changes, including erythema, hyperkeratosis, and, in some cases, blistering. Terms such as "PlayStation thumb", "Nintendo dermatitis", and "gamer's thumb" have been used to describe these conditions [51].

These dermatoses primarily affect the fingers and thumbs, reflecting patterns of repetitive motion and sustained contact. At a microenvironmental level, friction disrupts the stratum corneum, increases TEWL, and promotes localized inflammation. In children and adolescents, whose skin barrier may be more reactive, repeated low-grade mechanical trauma can lead to persistent irritation and, occasionally, secondary infection.

Pressure-Related Skin Changes

Sustained pressure from device use can result in transient or, less commonly, persistent skin changes. Prolonged placement of smartphones against the cheek during calls or continuous gripping of devices may lead to erythema, indentation, and localized discomfort. Although typically self-limited, repeated exposure may contribute to cumulative barrier disruption and increased susceptibility to irritant or inflammatory conditions.

In pediatric populations, behavioral patterns - such as extended gaming sessions or prolonged device use without breaks - may exacerbate pressure-related effects. While these changes are generally mild, their contribution to overall skin irritation should not be overlooked, particularly in individuals with underlying dermatologic conditions.

Acne Mechanica (Device-Related Acne)

Acne mechanica represents a clinically significant and increasingly recognized consequence of device use, particularly among adolescents. This condition arises from the combined effects of friction, occlusion, heat, and microbial contamination at sites of device contact, most commonly along the jawline and cheeks [52].

Occlusion and increased local humidity promote follicular hyperkeratinization and proliferation of *Cutibacterium *acnes, while friction induces microtrauma and inflammation. Additionally, mobile devices may serve as reservoirs for bacteria, sebum, and environmental debris, further exacerbating inflammatory processes. Adolescents are particularly susceptible due to hormonally driven increases in sebum production, creating a permissive environment for acne development. Emerging evidence suggests that high-frequency smartphone use correlates with increased acne severity, although causality remains to be fully established [53].

Irritant Contact Dermatitis

Irritant contact dermatitis associated with digital devices results from direct damage to the skin barrier rather than immune-mediated mechanisms. Prolonged exposure to heat, friction, and moisture at the device-skin interface can impair barrier integrity, leading to erythema, dryness, scaling, and burning sensations [54].

Individuals with pre-existing skin conditions, such as atopic dermatitis, are particularly vulnerable. In pediatric patients, the combination of frequent device use and an inherently sensitive skin barrier may amplify susceptibility. Unlike allergic contact dermatitis, irritant reactions may occur rapidly and are often dose-dependent, correlating with duration and intensity of exposure.

Collectively, these conditions reflect a localized disruption of the skin microenvironment at the device-skin interface, where friction, occlusion, heat, and microbial contamination converge to impair barrier integrity. This interface represents a unique and increasingly relevant dermatologic niche, particularly in pediatric populations characterized by high-frequency, prolonged digital engagement.

While most device-related dermatoses are mild and reversible, chronic or repetitive exposure may contribute to persistent skin barrier dysfunction and exacerbation of underlying dermatologic diseases. Recognition of these conditions is essential for clinicians, as early identification and targeted preventive strategies, such as reducing prolonged contact, maintaining device hygiene, and using protective barriers, can mitigate long-term consequences.

Blue light, screen exposure, and pigmentary disorders

Blue light, or HEV light (400-500 nm), represents a component of the visible spectrum with increasing relevance in dermatologic research. While sunlight remains the predominant source of blue light exposure, electronic devices such as smartphones, tablets, and computers contribute to cumulative, low-intensity exposure patterns characteristic of modern digital environments. The role of blue light in cutaneous pigmentation has emerged as a key area of investigation within digital photobiology.

Blue Light and Melanocyte Biology

Melanocytes are highly responsive to visible light stimuli, including blue light. Experimental studies have demonstrated that HEV light can induce melanogenesis through activation of photoreceptors such as opsin-3, leading to downstream signaling cascades that regulate melanin synthesis [55,56]. These pathways involve increased intracellular calcium flux and activation of transcription factors such as microphthalmia-associated transcription factor (MITF), which in turn upregulate melanogenic enzymes, including tyrosinase.

In addition to direct melanocyte activation, blue light exposure promotes oxidative stress within the skin microenvironment. Reactive oxygen species generated through photochemical interactions with endogenous chromophores further stimulate melanogenic pathways and contribute to pigmentary alterations [57]. These findings highlight a mechanistic link between digital light exposure and pigmentation at the cellular level.

Mechanisms of Melanogenesis and Pigmentary Change

Blue light-induced pigmentation differs from ultraviolet (UV)-mediated responses in both mechanism and clinical presentation. Unlike UVB-induced erythema and DNA damage, HEV light primarily induces immediate and persistent pigment darkening, particularly in melanin-rich skin. This process is mediated by oxidative signaling and melanocyte activation rather than direct DNA photodamage [56,58].

Importantly, blue light-induced pigmentation may be more sustained than UV-induced pigmentation in certain contexts, suggesting distinct regulatory pathways. Experimental dermatology studies have demonstrated that repeated exposure to visible light can lead to cumulative pigmentary changes, even at relatively low irradiance levels [55,57]. However, the magnitude of these effects under real-world digital exposure conditions remains an area of ongoing investigation.

Clinical Implications: Melasma and Post-Inflammatory Hyperpigmentation (PIH)

Emerging clinical evidence suggests that blue light exposure may contribute to the exacerbation of pigmentary disorders, particularly melasma and PIH. These conditions are characterized by dysregulated melanogenesis and are highly responsive to environmental triggers.

Visible light, including blue light, has been shown to induce pigmentation in patients with melasma, with studies demonstrating that HEV exposure can worsen pigmentation severity and prolong disease persistence [56,59]. Similarly, in individuals predisposed to PIH, oxidative stress and inflammatory signaling induced by light exposure may amplify melanocyte activity and delay the resolution of lesions.

Although most clinical studies have focused on solar visible light, these findings raise important considerations regarding cumulative exposure from digital sources, particularly in individuals with high baseline screen time.

Skin Phototype Differences

A consistent finding across experimental and clinical studies is the heightened susceptibility of darker skin phototypes (Fitzpatrick III-VI) to blue light-induced pigmentation. Increased baseline melanin content and heightened melanocyte responsiveness contribute to more pronounced and persistent pigmentary changes in these populations [55,56].

This differential response has important clinical implications, particularly in pediatric and adolescent populations in regions with higher proportions of darker skin phototypes. It underscores the need for tailored preventive strategies and further research into photoprotection against visible light.

Interaction With Ultraviolet Radiation

The effects of blue light should be interpreted within the broader context of solar radiation. Ultraviolet radiation remains the dominant driver of cutaneous photodamage, including DNA damage, erythema, and carcinogenesis. In comparison, the intensity of blue light emitted from digital devices is significantly lower, and its independent contribution to skin damage is less clearly defined.

However, blue light may act synergistically with ultraviolet radiation, contributing to cumulative oxidative stress and pigmentation. This interaction is particularly relevant in real-world settings, where individuals are exposed to multiple wavelengths simultaneously. Therefore, while UV protection remains the cornerstone of photoprotection, the role of visible light in pigmentary disorders is increasingly recognized.

Long-Term Implications and Clinical Relevance

The long-term dermatologic implications of chronic, low-level blue light exposure remain incompletely understood. Current evidence suggests that repeated exposure may contribute to cumulative pigmentary changes, particularly in susceptible individuals. However, the clinical significance of device-emitted blue light, relative to solar exposure, remains an area of active debate.

Overall, digital photobiology represents an emerging field that bridges environmental exposure and cutaneous response. While experimental data support a role for blue light in melanogenesis, further longitudinal and clinical studies are required to clarify its contribution to pigmentary disorders in real-world settings.

Indirect dermatologic effects of digital lifestyle

The rapid integration of digital technologies into daily life has profoundly reshaped behavioral patterns, particularly among children and adolescents. Beyond direct device-skin interactions, digital exposure exerts significant indirect effects on dermatologic health through its influence on sleep, stress, diet, physical activity, and environmental exposure. These interconnected lifestyle factors form the basis of “lifestyle dermatology” in the digital age, highlighting the complex pathways through which behavior mediates cutaneous outcomes.

Sleep Disruption and Circadian Dysregulation

Prolonged digital device use, particularly during evening hours, has been associated with delayed sleep onset and reduced sleep duration in pediatric populations [60,61]. Disruption of circadian rhythms can impair cutaneous homeostasis, as key processes such as epidermal repair, barrier recovery, and cellular turnover are regulated in a time-dependent manner. Sleep disruption may impair normal barrier recovery and skin hydration [62,63].

Although the molecular mechanisms underlying circadian regulation have been discussed previously, it is important to emphasize that behavioral sleep disruption represents a key mediator linking digital exposure to dermatologic outcomes. Altered sleep patterns may also influence the skin microbiome; however, current evidence remains limited and requires further validation [64].

Stress and the Neurocutaneous Axis

Digital engagement, particularly through social media platforms, has been associated with increased psychological stress, anxiety, and appearance-related concerns among adolescents. Chronic stress activates the hypothalamic-pituitary-adrenal (HPA) axis, leading to elevated cortisol levels, which exert multiple effects on the skin, including impaired wound healing, altered collagen synthesis, and increased sebaceous gland activity [65].

The neurocutaneous axis provides a framework for understanding these interactions, emphasizing the bidirectional relationship between the nervous system and the skin. In conditions such as acne, psoriasis, and atopic dermatitis, stress can exacerbate disease severity, creating a feedback loop in which dermatologic symptoms further contribute to psychological distress. Social media-driven comparison and cyberbullying may further amplify this cycle in vulnerable populations [66].

Sedentary Lifestyle and Metabolic Dysregulation

High levels of screen time are closely associated with sedentary behavior, reduced physical activity, and increased risk of obesity and metabolic syndrome [67,68]. These metabolic alterations are increasingly recognized as modifiers of dermatologic disease. Insulin resistance and hyperinsulinemia can promote sebaceous gland activity and follicular hyperkeratinization through pathways involving insulin-like growth factor-1 and mTORC1 signaling, contributing to acne pathogenesis [69,70].

In addition, systemic inflammation associated with metabolic dysregulation may exacerbate chronic inflammatory skin conditions. These findings underscore the importance of considering dermatologic health within a broader metabolic context, particularly in digitally engaged pediatric populations.

Diet and Acne

Digital lifestyles are often associated with unhealthy dietary patterns, including increased consumption of high glycemic index foods and ultra-processed diets [71]. Such dietary habits have been associated with acne development and severity through their effects on insulin signaling and hormonal regulation. Elevated insulin levels stimulate androgen production and increase insulin-like growth factor-1, promoting sebocyte proliferation and follicular hyperkeratinization [72].

Clinical studies have demonstrated that low glycemic load diets may reduce acne severity, supporting the role of diet as a modifiable factor in dermatologic health [73]. While diet alone does not fully account for acne pathogenesis, its interaction with other lifestyle factors, such as sleep and stress, contributes to a multifactorial disease model.

Psychodermatologic Interactions

Psychodermatology represents the complex interplay between psychological processes and cutaneous disease, mediated through neuroendocrine, immune, and behavioral pathways [74]. In the digital era, this relationship has become increasingly relevant, as social media and online environments exert profound influences on mental health, self-perception, and skin-related behaviors among adolescents.

Social media platforms play a pivotal role in shaping skincare practices and dermatologic perceptions. Adolescents are frequently exposed to curated beauty standards and "dermatology trends", including do-it-yourself (DIY) skincare regimens, unregulated cosmetic use, and influencer-driven product recommendations. These practices often lack scientific validation and may lead to inappropriate or excessive use of topical agents, resulting in irritant dermatitis, barrier disruption, or exacerbation of underlying conditions. The rapid dissemination of such trends is facilitated by the viral nature of online content, where misinformation can spread widely and influence health behaviors [63].

Online dermatology misinformation represents a growing concern within this context. Social media platforms frequently host unverified claims regarding acne treatments, pigmentation therapies, and “natural remedies,” which may delay appropriate medical care or promote harmful practices. Studies analyzing health-related misinformation on social media have demonstrated that inaccurate content can achieve substantial engagement and dissemination, particularly when it aligns with user beliefs or appears easily actionable [66].

The psychological impact of social media extends beyond behavioral influences to affect body image and self-esteem. Adolescents are particularly vulnerable to appearance-based comparison, which may contribute to body dissatisfaction and heightened awareness of perceived skin imperfections. This phenomenon is closely linked to dermatologic anxiety, wherein minor or transient skin changes are perceived as significant cosmetic concerns. Such anxiety may exacerbate conditions like acne or atopic dermatitis through stress-mediated pathways, reinforcing a bidirectional cycle between psychological distress and skin disease [75].

Skin picking disorder (dermatillomania), a recognized psychodermatologic condition, may also be influenced by digital behaviors. Increased screen time and heightened visual attention to facial appearance, particularly during video calls or social media use, can promote repetitive skin-focused behaviors. These behaviors may result in excoriations, secondary infections, and PIH, further contributing to psychological distress [68].

Importantly, psychodermatologic interactions are inherently bidirectional. Dermatologic conditions themselves can significantly impair quality of life and mental health, increasing the risk of anxiety, depression, and social withdrawal. In digitally connected environments, this burden may be amplified by online visibility, cyberbullying, and social comparison. Consequently, adolescents with visible skin conditions may experience disproportionate psychosocial impact, further influencing lifestyle behaviors such as sleep, diet, and treatment adherence [75].

Collectively, these observations highlight the role of digital environments in shaping psychodermatologic outcomes. Social media-driven behaviors, misinformation, and psychological stressors converge to influence both the perception and progression of skin disease. Recognizing these factors is essential for clinicians, as effective management of dermatologic conditions in pediatric populations increasingly requires addressing not only biological mechanisms but also the psychological and behavioral context of digital life.

Reduced Outdoor Activity and Vitamin D

Increased screen time is associated with reduced outdoor activity and decreased sunlight exposure. This behavioral shift has been associated with vitamin D deficiency in children and adolescents [76]. Vitamin D plays a role in immune regulation and skin barrier function, and deficiency has been associated with inflammatory skin conditions such as atopic dermatitis and psoriasis.

However, these associations should be interpreted cautiously, as causality remains complex and multifactorial. Adequate outdoor activity remains important not only for vitamin D synthesis but also for overall physical and mental well-being.

**Figure 3:**
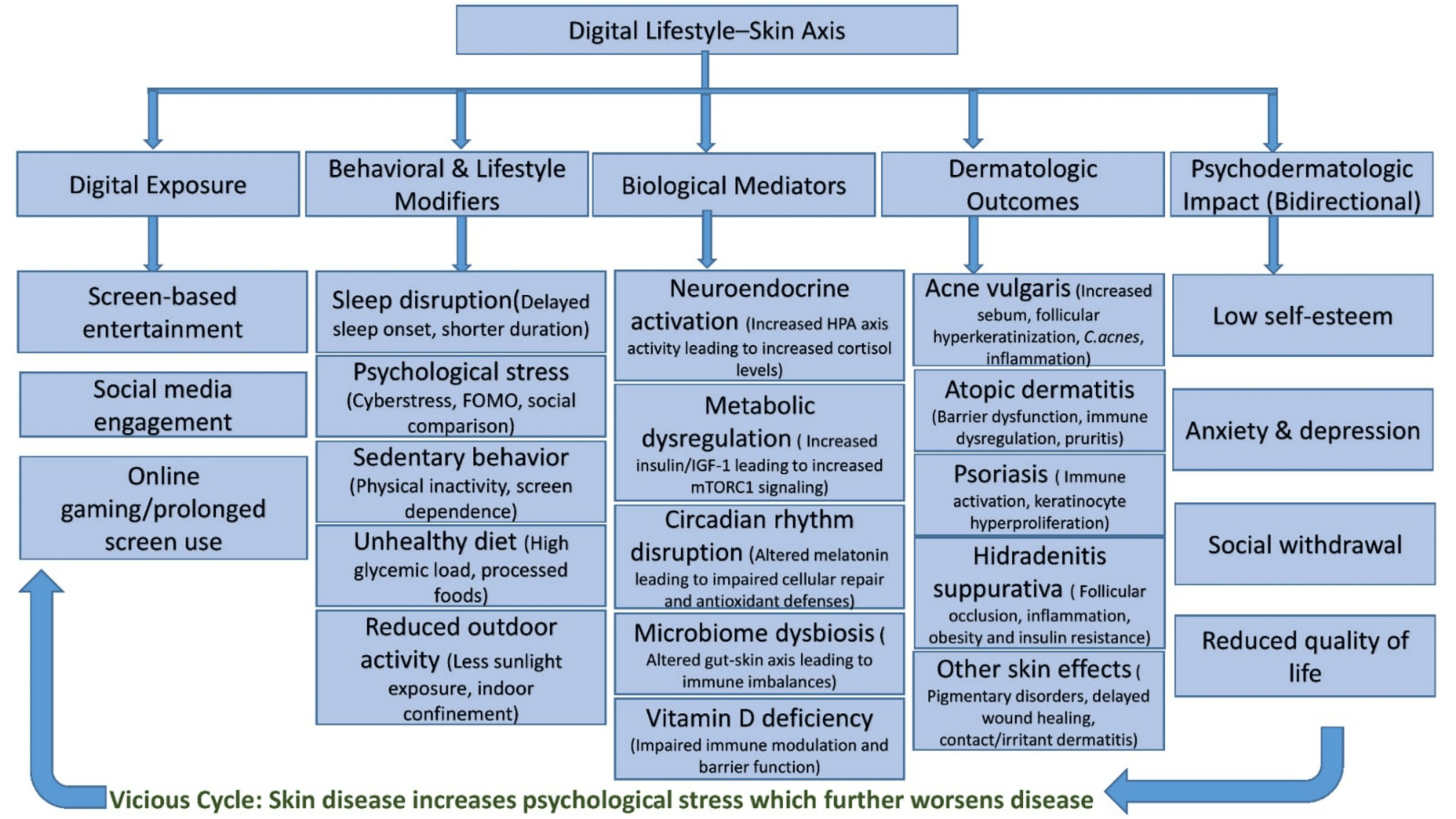
Digital Lifestyle-Skin Axis: Behavioral and Psychodermatologic Pathways in Children and Adolescents

These interconnected lifestyle factors highlight the complex, indirect pathways through which digital exposure influences dermatologic health in pediatric populations. Rather than acting independently, sleep disruption, stress, diet, and sedentary behavior converge to modulate skin barrier function, immune responses, and inflammatory pathways. Understanding these relationships is essential for developing holistic, preventive strategies that address not only direct environmental exposures but also the broader behavioral context of digital life. A systems-level model illustrating the digital lifestyle-skin axis is presented in Figure 3, and the key indirect lifestyle factors linking digital exposure to dermatologic outcomes are summarized in Table 2.

**Table 2:** Indirect Lifestyle Factors Linking Digital Exposure to Dermatologic Outcomes in Children and Adolescents Abbreviations: TEWL: transepidermal water loss; HPA axis: hypothalamic-pituitary-adrenal axis; CRH: corticotropin-releasing hormone; IGF-1: insulin-like growth factor 1; mTORC1: mechanistic target of rapamycin complex 1; UV-B: ultraviolet B radiation

Lifestyle Factor	Digital Exposure Driver	Underlying Biological Mechanism	Dermatologic Impact	Key References
Sleep Disruption	Late-night screen use; blue light exposure	Circadian rhythm disruption; ↓ melatonin; impaired keratinocyte proliferation; ↑ cortisol	Impaired barrier repair; ↑ TEWL; atopic dermatitis flares; delayed wound healing	[17,18,60,62]
Psychological Stress	Social media comparison; cyberbullying; excessive engagement	HPA axis activation; ↑ cortisol; CRH-mediated sebocyte activation; neuroinflammation	Acne exacerbation; psoriasis flares; impaired wound healing; psychodermatologic disorders	[66,74,75]
Sedentary Behavior & Obesity	Prolonged screen time; reduced physical activity	Insulin resistance; ↑ IGF-1; mTORC1 activation; systemic inflammation	Acne vulgaris; hidradenitis suppurativa; psoriasis exacerbation	[3,67,69,70]
Dietary Changes (High Glycemic Load)	Screen-associated snacking; ultra-processed food intake	Hyperinsulinemia; ↑ IGF-1; androgen-mediated sebogenesis; follicular hyperkeratinization	Increased acne severity; inflammatory dermatoses	[20,71,72]
Reduced Outdoor Activity	Screen time replacing outdoor play	↓ UV-B exposure → ↓ vitamin D synthesis; altered immune modulation	Atopic dermatitis; psoriasis; impaired skin immunity	[22,76]
Altered Skin Microbiome	Poor sleep; sedentary lifestyle; occlusion from device use	Dysbiosis; altered microbial diversity; impaired innate immunity	Acne; eczema; increased susceptibility to infection	[64]
Psychodermatologic Behaviors	Increased self-image awareness; video calls; social media	Behavioral reinforcement loops; compulsive skin picking; stress–skin axis activation	Acne excoriée; post-inflammatory hyperpigmentation; scarring	[68,75]

Prevention strategies and public health interventions

The increasing recognition of digital exposure as a determinant of dermatologic health necessitates the development of structured prevention strategies that integrate clinical guidance with public health interventions. A “dermatologic digital hygiene framework” emphasizes behavioral modification, environmental control, and education to mitigate both direct and indirect skin-related effects of digital lifestyles.

Screen-Time Recommendations and Behavioral Regulation

Screen-time moderation remains a cornerstone of preventive strategies. Current recommendations from the AAP and the WHO emphasize age-specific guidance rather than uniform limits. Infants younger than 18-24 months should avoid screen exposure except for video communication, while children aged two to five years should be limited to approximately one hour of high-quality content daily [61,77]. For older children and adolescents, emphasis is placed on maintaining balanced use that does not interfere with sleep, physical activity, and psychosocial well-being [77].

Behavioral strategies, such as establishing screen-free periods (e.g., before bedtime) and device-free zones (e.g., bedrooms and mealtimes), have demonstrated benefits in improving sleep quality and reducing circadian disruption [78]. Additionally, structured screen breaks, such as the “20-20-20 rule,” originally developed to reduce digital eye strain, may indirectly help minimize prolonged device exposure, sedentary behavior, and associated physiologic stress [79].

Blue Light Protection Strategies

Given the role of blue light in circadian disruption and its potential cutaneous effects, several protective strategies have been proposed. These include the use of night mode settings, blue light-filtering applications, and screen filters that shift emitted wavelengths toward longer spectra [80]. Behavioral interventions, such as reducing screen exposure before bedtime, may support healthier sleep patterns and overall digital wellness [78,81].

Although these interventions are widely promoted for digital wellness and sleep hygiene, direct clinical dermatologic benefits remain insufficiently validated, and current evidence regarding their effectiveness in preventing skin damage is limited and evolving [42]. Nevertheless, these measures are generally considered low-risk and may contribute to broader healthy digital-use practices.

Device Hygiene and Skin Contact Prevention

At the device-skin interface, preventive measures should focus on minimizing mechanical and microbial insult. Regular cleaning of smartphones and tablets reduces the accumulation of sebum, bacteria, and environmental contaminants that may exacerbate acne and irritant dermatitis. The use of protective cases and hands-free accessories (e.g., earphones or speaker mode) can reduce prolonged skin contact and friction-related injury.

In pediatric populations, where prolonged and repetitive device use is common, these measures are particularly relevant for preventing acne mechanica, contact dermatitis, and frictional dermatoses. Education regarding the avoidance of direct contact with metallic surfaces may further reduce the risk of nickel-induced allergic reactions.

Pediatric Dermatology Counseling

Clinicians play a critical role in integrating digital exposure counseling into routine dermatologic care. Counseling should extend beyond topical treatments to include lifestyle assessment, encompassing screen habits, sleep patterns, diet, and psychosocial factors. Adolescents with acne, atopic dermatitis, or pigmentary disorders may particularly benefit from individualized guidance on digital behaviors.

Importantly, counseling should adopt a balanced approach, acknowledging the functional role of digital devices while promoting moderation and safe use. Incorporating digital hygiene into anticipatory guidance may enhance adherence and improve long-term outcomes.

Role of Parents, Schools, and Public Health Systems

Effective prevention requires a multi-level approach involving families, educational institutions, and public health systems. Parents play a central role in modeling healthy digital behaviors, establishing boundaries, and supervising media use. Co-viewing and engagement with children's digital activities can enhance awareness and reduce harmful exposure patterns [61].

Schools can contribute by integrating digital wellness education into curricula, promoting screen breaks, and encouraging outdoor activities. Public health initiatives should focus on raising awareness regarding the dermatologic implications of digital lifestyles, particularly in high-risk pediatric populations.

Technology Design and Future Directions

Emerging strategies also involve technology-based solutions, including improved screen design, adaptive brightness controls, and reduced blue light emission. Ergonomic considerations, such as maintaining appropriate viewing distance, posture, and ambient lighting, may further reduce physical and dermatologic strain associated with prolonged device use [79].

Future research should focus on developing evidence-based guidelines specific to dermatologic outcomes, as current recommendations are largely extrapolated from sleep and ophthalmologic data. As digital exposure continues to evolve, interdisciplinary collaboration between dermatology, pediatrics, and public health will be essential.

Collectively, these strategies form a comprehensive dermatologic digital hygiene framework that addresses both behavioral and environmental determinants of skin health. By combining individual-level interventions with broader public health measures, it is possible to mitigate the dermatologic impact of digital exposure while preserving the benefits of technology in pediatric populations.

## Conclusions

The expanding integration of digital technologies into the daily lives of children and adolescents represents a novel and multifaceted environmental determinant of dermatologic health. This state-of-the-art review highlights that digital exposure exerts its effects through a convergence of direct photobiological, mechanical, and thermal influences, as well as indirect behavioral and psychosocial pathways that collectively disrupt skin barrier integrity, circadian regulation, immune function, and neuroendocrine balance. Pediatric skin, characterized by developmental immaturity and heightened biological sensitivity, appears particularly vulnerable to these cumulative exposures, manifesting in a spectrum of conditions ranging from acne and atopic dermatitis to pigmentary disorders and emerging device-related dermatoses. Importantly, the interplay between digital behaviors and psychodermatologic factors underscores a bidirectional relationship that may perpetuate disease burden and impact quality of life. While current evidence provides important mechanistic and clinical insights, much of the available literature remains observational, experimental, or indirect in nature and is limited by heterogeneity and a lack of longitudinal real-world pediatric data. Future research should focus on elucidating dose-response relationships, long-term outcomes, and population-specific vulnerabilities. In parallel, the development of evidence-based "digital dermatologic hygiene" strategies - integrating clinical guidance, behavioral interventions, and public health measures - will be essential to mitigate risks while preserving the benefits of digital engagement in this increasingly connected generation.
